# Supplementary material for: α7 nicotinic acetylcholine receptor upregulation by anti-apoptotic Bcl-2 proteins
Source: Nat Commun. 2019 Jun 21;10:2746. doi: 10.1038/s41467-019-10723-x (PMC6588605; doi:10.1038/s41467-019-10723-x)
Supplement: Supplementary file 1 — Supplementary Information [file 41467_2019_10723_MOESM1_ESM.pdf]

α7 nicotinic acetylcholine receptor upregulation by anti-apoptotic Bcl-2 proteins

Supplementary Information

G. Brent Dawe, Hong Yu, Shenyan Gu, Alissa N. Blackler, Jose A. Matta, Edward R. Siuda, Elizabeth B. Rex, David S. Bredt

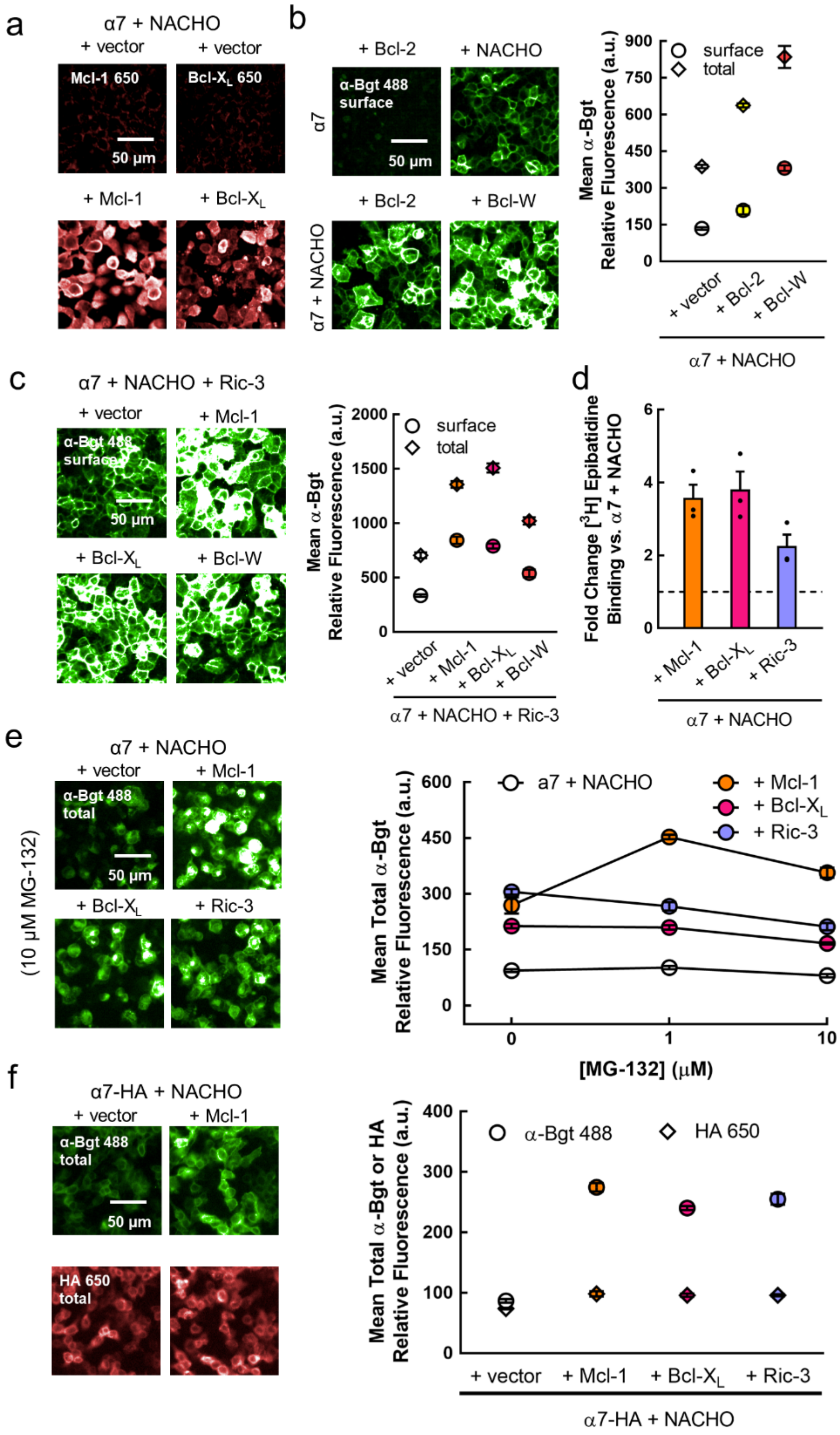

Supplementary Figure 1. *Legend on following page.*

Supplementary Figure 1. Assembly of α7 nAChRs is enhanced by anti-apoptotic Bcl-2 family proteins.
a Fluorescent labeling of Bcl-2 family proteins in permeabilized HEK293T cells transfected with cDNAs encoding α7 and NACHO, along with Mcl-1 or Bcl-X_L, at a 1:3:5 respective ratio.
b Fluorescent α-Bgt labeling of non-permeabilized HEK293T cells cotransfected with cDNAs encoding α7 and NACHO, along with Bcl-2 or Bcl-W at a 1:3:4 cDNA ratio (left). Quantification of fluorescence intensity from α7 labeling by α-Bgt in various conditions (right; $n = 5$). Increases in surface and total staining with Bcl-2 and Bcl-W coexpression were significant ($p < 0.02$).
c Fluorescent α-Bgt labeling of non-permeabilized HEK293T cells cotransfected with cDNAs encoding α7, NACHO, and Ric-3, along with empty vector, Mcl-1, Bcl-X_L, or Bcl-W at a 1:3:4:4 cDNA ratio (left). Quantification of fluorescence intensity from α7 labeling by α-Bgt in various conditions (right; $n = 5$). Increases in surface and total staining with Bcl-2 protein coexpression were significant ($p < 0.003$).
d Fold change in [^{3}H]epibatidine binding to HEK293T cell membranes cotransfected with cDNAs encoding α7 and NACHO, along with other proteins indicated at a 1:3:5 respective ratio. Data are from experiments performed after three independent transfections.
e Fluorescent α-Bgt labeling of permeabilized HEK293T cells cotransfected with cDNAs encoding α7 and NACHO, along with Mcl-1, Bcl-X_L, Ric-3, or empty vector at a 1:3:4 cDNA ratio, incubated for 24 h before fixation with the cell-permeable proteasome inhibitor MG-132 (left). Quantification of fluorescence intensity from α7 labeling by α-Bgt (right; $n = 5$). Increases in total staining with Bcl-2 protein or Ric-3 coexpression remained significant in 10 µM MG-132 ($p < 1e^{-4}$).
f Fluorescent α-Bgt and HA labeling of permeabilized HEK293T cells cotransfected with cDNAs encoding HA-tagged α7 and NACHO, along with empty vector, Mcl-1, Bcl-X_L, or Ric-3 at a 1:3:4 cDNA ratio (left). Quantification of fluorescence intensity from α7 labeling in various conditions (right; $n = 4$, HA, or $n = 5$, α-Bgt).
All data are means ± SEM; *P*-values from two-sample *t*-test. For all α-Bgt labeling experiments, the same cDNA transfection conditions were repeated at different passage numbers and yielded similar results.

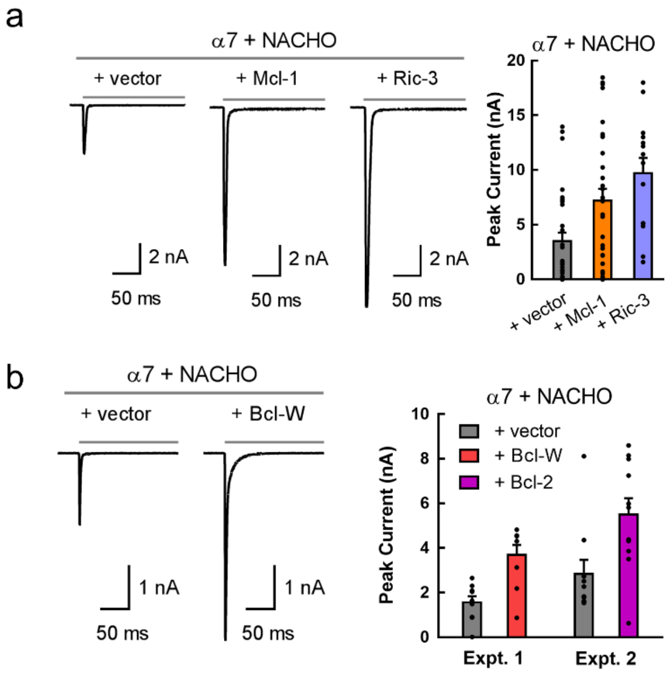

Supplementary Figure 2. Functional expression of α7 nAChRs in the presence of NACHO is further enhanced by several anti-apoptotic Bcl-2 family proteins.

a, b Representative ACh-evoked whole-cell current responses elicited from HEK293T cells expressing α7 and NACHO, along with Bcl-2 members or Ric-3 proteins indicated. For panel **a**, cDNAs were transfected at a 3:1:10 ratio of α7, NACHO, and Mcl-1 or Ric-3, respectively, and 3 mM ACh was applied to cells. In contrast, for a separate set of experiments shown in panel **b**, equal cDNA amounts were transfected, while 1 mM ACh was utilized. Summary graphs of agonist-evoked peak currents for these same experiments in panel **a** (*n* = 31, α7 + NACHO; 32, + Mcl-1; 14, + Ric-3) and panel **b** (*n* = 9, α7 + NACHO Expt. 1; 9, + Bcl-W; 10, α7 + NACHO Expt. 2; 11, + Bcl-2) are also shown. Increases in peak current response with Ric-3 or Bcl-2 protein coexpression were significant ($p < 0.01$).

All data are means ± SEM; *P*-values from two-sample *t*-test.

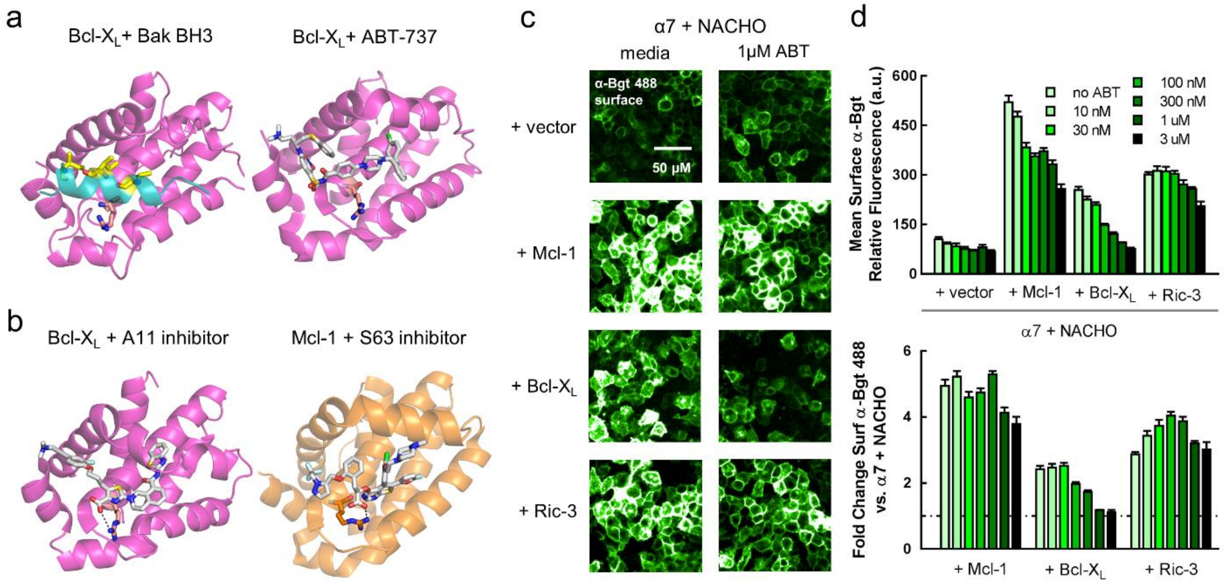

Supplementary Figure 3. Chemical inhibitors of Bcl-2 family proteins reduce Bcl-2-mediated upregulation of α7 nAChR expression.

a NMR structure of Bcl-X_L bound by the BH3 peptide of pro-apoptotic Bcl-2 member Bak (left, PDB: 1BXL;[1]) and X-ray crystal structure of Bcl-X_L bound by the Bcl-2 inhibitor ABT-737 (right, PDB: 2YXJ;[2]).

b Crystal structures of Bcl-X_L (left) and Mcl-1 (right), respectively bound by the selective chemical inhibitors A-1155463 (A11; PDB: 4QVX;[3]) and S-63845 (S63; PDB: 5LOF;[4]).

c Fluorescent α-Bgt labeling of non-permeabilized HEK293T cells cotransfected with cDNAs encoding α7 and NACHO, along with other proteins, at a 1:3:4 (α7:NACHO:other) ratio. Cells in the right column were incubated with the Bcl-2 inhibitor ABT-737 for 24 h.

d Quantification of α7 labeling by α-Bgt at various ABT-737 concentrations, presented as mean fluorescence intensity (top) and the fold change in fluorescence intensity relative to α7 and NACHO (expressed alone) at the same inhibitor concentration (bottom; $n = 5$). In 3 µM ABT-737, fluorescence intensity was still significantly enhanced by Mcl-1 and Ric-3 ($p < 0.001$), but not Bcl-X_L ($p = 0.32$), though all were effective without ABT-737 ($p < 1e^{-8}$). The same cDNA combinations were transfected at different passage numbers and yielded similar results.

All data are means ± SEM; *P*-values from two-sample *t*-test.

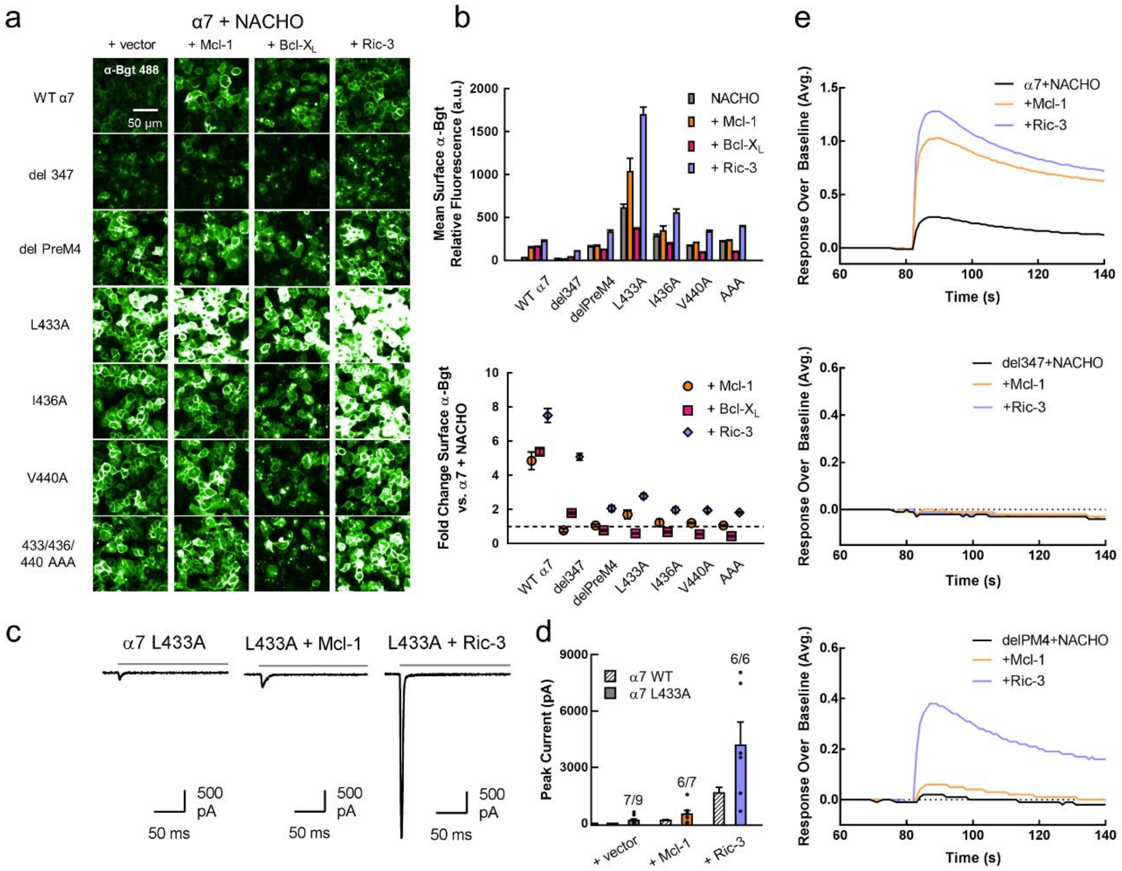

Supplementary Figure 4. α-Bgt labeling and functional expression of α7 pre-M4 region mutants is insensitive to Bcl-2 coexpression.

a Fluorescent α-Bgt labeling of non-permeabilized HEK293T cells cotransfected with cDNAs encoding wildtype or mutant α7 and NACHO, along with other proteins indicated, at a 1:3:4 respective ratio.

b Quantification of surface α-Bgt labeling of various α7 mutants, presented as mean fluorescence intensity (top) and the fold change in fluorescence intensity relative to α7 and NACHO expressed alone (bottom; n = 5). The same cDNA combinations were transfected at different passage numbers and yielded similar results.

c Representative whole-cell current responses evoked by 3 mM ACh from HEK293T cells expressing α7 L433A, alone or with Mcl-1 or Ric-3.

d Summary graph of agonist-evoked peak currents for experiments in panel **c** (n = 9, L433A; 7, + Mcl-1; 6, + Ric-3) with the number of responsive cells indicated. Coexpression of α7 L433A with Ric-3 (p = 0.02), but not Mcl-1 (p = 0.15), significantly increased peak current response. Data from wildtype α7 receptors coexpressed with the same additional proteins (from Fig. 1) is also shown at left for each condition.

e FLIPR traces showing 100 μM Nic-evoked Ca^{2+} flux through HEK293T cells cotransfected with cDNAs encoding wildtype (top) or mutant (middle and bottom) α7 and NACHO, along with Mcl-1, Ric-3, or empty vector at a 1:1:5 respective ratio. The traces are averages of individual responses from separate wells (n = 14), and response amplitudes are relative to the background fluorescence signal prior to agonist application.

All data are means ± SEM; P-values from two-sample t-test.

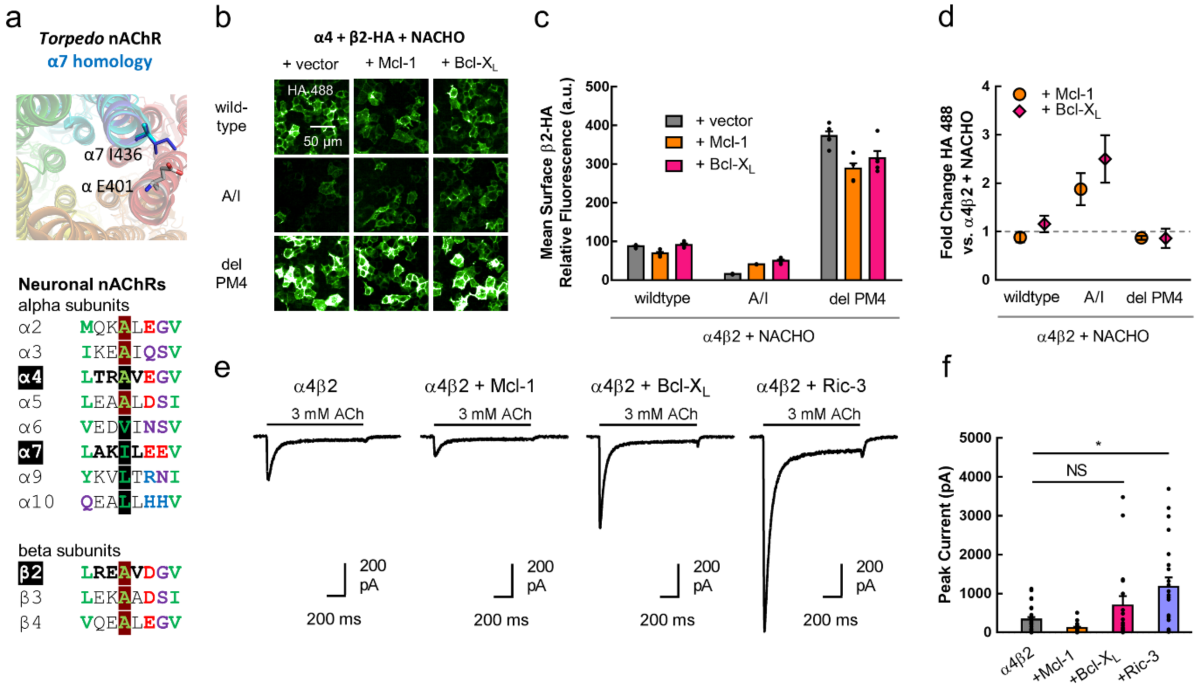

Supplementary Figure 5. α4β2 nAChRs are largely insensitive to regulation by Bcl-2 proteins.

a Homology model of the α7 pre-M4 helix aligned to the *Torpedo marmorata* muscle nAChR (PDB: 2BG9;[5]), for which part of the TM3-TM4 intracellular loop is resolved (top). A sequence alignment of human neuronal nAChR subunits reveals that the position equivalent to isoleucine 436 in α7 typically contains an alanine residue in assembly competent subunits like α4 and β2 (bottom).

b Fluorescent HA labeling of non-permeabilized HEK293T cells cotransfected with cDNAs encoding α4, β2-HA, and NACHO, along with several Bcl-2 proteins, at a 1:1:2:5 respective ratio.

c, d Quantification of surface HA labeling of wildtype and mutant α4β2-HA receptors, presented as fluorescence intensity (n = 5) and the fold change in fluorescence intensity from Bcl-2 coexpression, relative to α4β2 and NACHO (**d**). Data in panel **d** are taken from independent experiments (n = 4) where Mcl-1 and Bcl-X_L increased surface staining significantly ($p = 0.003$, Mcl-1; $p = 0.001$, Bcl-X_L; paired t-test) for only the A/I mutant receptors.

e Representative whole-cell current responses elicited from HEK293T cells cotransfected with cDNAs encoding α4 and β2 subunits, alone or with Mcl-1, Bcl-X_L, or Ric-3 at a 1:1:5 respective ratio.

f Summary graph of agonist-evoked peak currents for transfections described in panel **e** (*$p < 0.01$; n = 26, α4β2; 18, + Mcl-1; 18, + Bcl-X_L; 22, + Ric-3). Coexpression with Ric-3, but not Bcl-X_L ($p = 0.16$, two-sample t-test), yielded a significant increase in current amplitude.

All data are means ± SEM.

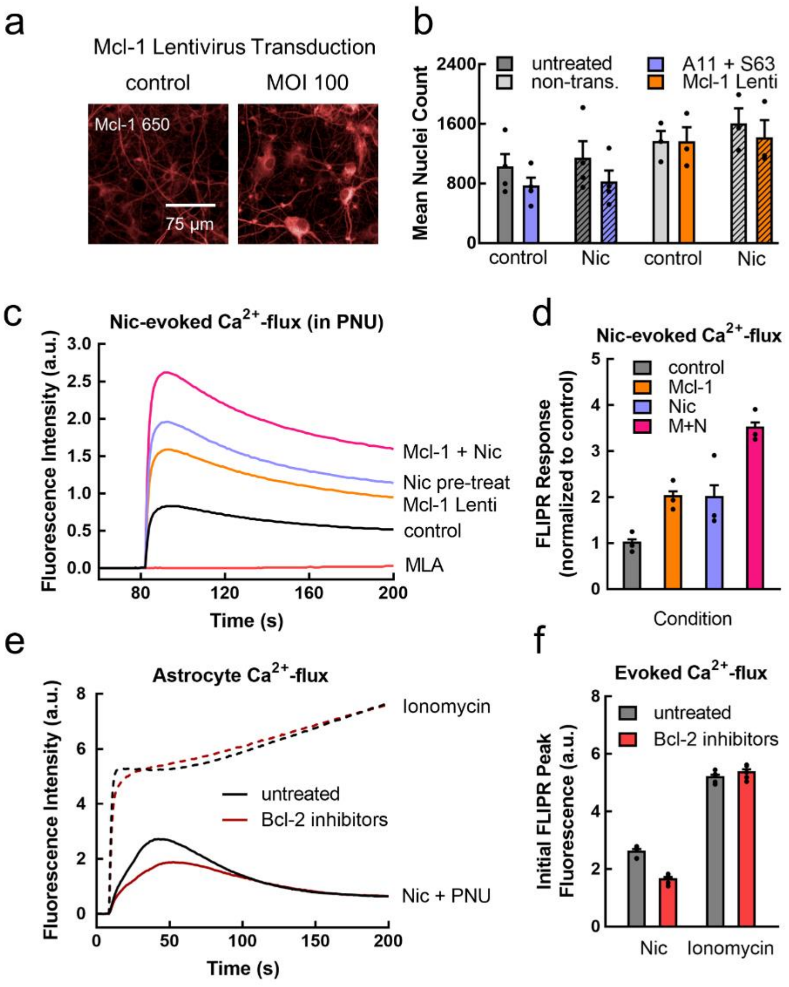

Supplementary Figure 6. *Legend on following page.*

Supplementary Figure 6. Bcl-2 proteins regulate α7 nAChR activity in hippocampal neurons and astrocytes.
a Fluorescent labeling of Mcl-1 on rat hippocampal neurons (DIV 20) transduced with lentivirus encoding human Mcl-1 at DIV7.
b Mean nuclei count per field obtained from DAPI staining of hippocampal cultures. For control and nicotine-treated populations incubated with Bcl-2 inhibitors, a modest, but significant reduction in nuclei count was observed ($p = 0.04$, paired *t*-test), though viable cells were selected for surface α-Bgt and GluA1 quantification (see Experimental Procedures). Mcl-1 lentivirus transduction at MOI 100 did not significantly affect nuclei number versus the non-transduced condition ($p = 0.23$, paired *t*-test).
c FLIPR traces showing 100 µM nicotine-evoked Ca^{2+} flux through α7 nAChRs expressed on rat hippocampal neurons in the presence of the positive allosteric modulator PNU-120596. Responses are shown for Mcl-1 lentivirus-transduced and/or nicotine pre-treated neurons, normalized to the background fluorescence signal. As a negative control, responses were blocked by the nAChR antagonist methyllycaconitine (MLA) at 100 nM.
d Summary graph of the peak FLIPR response from experiments described in panel **c**, normalized to the response of non-transduced/untreated neurons. Data are averaged from three experiments on different cultures. Significant increases in peak response were observed for Mcl-1 transduced neurons (MOI 100) with and without nicotine pre-treatment ($p = 0.03$, Nic; $p = 0.01$, control, paired one-tail *t*-test).
e Representative FLIPR traces showing Ca^{2+} flux through astrocyte cell membranes in response to 25 µM nicotine and 5 µM PNU-120596 or 5 µM ionomycin. Some cells were treated for 96 h with the Bcl-X_L inhibitor A-1155463 (30 nM), Mcl-1 inhibitor S-63845 (30 nM), and general Bcl-2 protein inhibitor ABT-737 (30 nM) prior to the assay. Ionomycin was used as a control to elicit maximal Ca^{2+} flux based on the number of cells present.
f Summary graph of the initial peak FLIPR response (at 45 s) from experiments described in panel **e** (n = 5). Bcl-2 inhibitors induced a significant decrease in the nicotine, but not ionomycin, evoked response ($p < 1e^{-4}$, nicotine; $p = 0.28$, ionomycin; two-sample *t*-test).
All data are means ± SEM.

Supplementary Table 1.

Effect of Bcl-2 family protein and/or Ric-3 coexpression with α7 nAChR & NACHO on α-Bgt labeling of α7 pentamers and HA labeling of α7 subunits.

Condition/ cDNA	cDNA Ratio	Fluorescence Intensity (a.u.)	Fold Change vs. α7 + NACHO	n Value
surface α-Bgt; Fig. 1b				
α7 + NACHO + vector	1:3:5	62.66 ± 8.96		5
α7 + NACHO + Mcl-1	1:3:5	265.32 ± 19.31	4.23 ± 0.31	5
α7 + NACHO + Bcl-X_L	1:3:5	180.80 ± 9.20	2.89 ± 0.15	5
α7 + NACHO + Ric-3	1:3:5	285.60 ± 13.77	4.56 ± 0.22	5
total α-Bgt; Fig. 1b				
α7 + NACHO + vector	1:3:5	195.14 ± 15.79		5
α7 + NACHO + Mcl-1	1:3:5	680.60 ± 50.59	3.49 ± 0.26	5
α7 + NACHO + Bcl-X_L	1:3:5	473.62 ± 32.62	2.43 ± 0.17	5
α7 + NACHO + Ric-3	1:3:5	428.72 ± 32.69	2.20 ± 0.17	5
surface α-Bgt; Sup. Fig. 1b				
α7 + NACHO + vector	1:3:4	134.26 ± 5.58		5
α7 + NACHO + Bcl-2	1:3:4	209.17 ± 17.79	1.56 ± 0.13	5
α7 + NACHO + Bcl-W	1:3:4	381.72 ± 11.44	2.84 ± 0.09	5
α7 + NACHO + Ric-3	1:3:4	446.48 ± 10.49	3.33 ± 0.08	5
total α-Bgt; Sup. Fig. 1b				
α7 + NACHO + vector	1:3:4	388.10 ± 6.81		5
α7 + NACHO + Bcl-2	1:3:4	637.91 ± 8.14	1.64 ± 0.02	5
α7 + NACHO + Bcl-W	1:3:4	835.85 ± 44.97	2.15 ± 0.12	5
α7 + NACHO + Ric-3	1:3:4	898.67 ± 18.44	2.32 ± 0.05	5
surface α-Bgt; Sup. Fig. 1c				
α7 + NACHO + Ric-3 + vector	1:3:4:4	336.39 ± 11.73		5
α7 + NACHO + Ric-3 + Mcl-1	1:3:4:4	843.39 ± 34.90	2.51 ± 0.10	5
α7 + NACHO + Ric-3 + Bcl-X_L	1:3:4:4	790.67 ±23.37	2.35 ± 0.07	5
α7 + NACHO + Ric-3 + Bcl-W	1:3:4:4	539.70 ±35.81	1.60 ± 0.11	5
total α-Bgt; Sup. Fig. 1c				
α7 + NACHO + Ric-3 + vector	1:3:4:4	706.29 ±27.03		5
α7 + NACHO + Ric-3 + Mcl-1	1:3:4:4	1357.30 ± 29.79	1.92 ± 0.04	5
α7 + NACHO + Ric-3 + Bcl-X_L	1:3:4:4	1509.73 ± 42.51	2.14 ± 0.06	5
α7 + NACHO + Ric-3 + Bcl-W	1:3:4:4	1022.03 ± 34.44	1.45 ±0.05	5
Continued on next page.				

total α-Bgt; Sup. Fig. 1f				
α7-HA + NACHO + vector	1:3:4	86.94 ± 3.17		5
α7-HA + NACHO + Mcl-1	1:3:4	274.85 ± 7.14	3.16 ± 0.08	5
α7-HA + NACHO + Bcl-X_L	1:3:4	240.52 ± 3.43	2.77 ± 0.04	5
α7-HA + NACHO + Ric-3	1:3:4	255.22 ± 9.03	2.94 ± 0.10	5
total HA; Sup. Fig. 1f				
α7-HA + NACHO + vector	1:3:4	74.01 ± 1.55		4
α7-HA + NACHO + Mcl-1	1:3:4	98.36 ± 4.41	1.33 ± 0.06	4
α7-HA + NACHO + Bcl-X_L	1:3:4	96.28 ± 2.76	1.30 ± 0.04	4
α7-HA + NACHO + Ric-3	1:3:4	96.20 ± 1.85	1.30 ± 0.02	4
surface α-Bgt; Fig. 2b				
α7 + NACHO ; 0 nM S63	1:3	39.95 ± 2.10		10
α7 + NACHO + Mcl-1; 0 nM S63	1:3:4	197.35 ± 7.40	4.94 ± 0.19	10
α7 + NACHO + Mcl-1; 10 nM S63	1:3:4	163.36 ± 4.63	3.62 ± 0.10	5
α7 + NACHO + Mcl-1; 100 nM S63	1:3:4	90.67 ± 3.65	2.34 ± 0.09	5
α7 + NACHO + Mcl-1; 1 µM S63	1:3:4	82.39 ± 7.05	2.34 ± 0.20	5
surface α-Bgt; Fig. 2b				
α7 + NACHO ; 0 nM A11	1:3	40.22 ± 1.49		10
α7 + NACHO + Bcl-X_L; 0 nM A11	1:3:4	142.95 ± 8.09	3.55 ± 0.20	10
α7 + NACHO + Bcl-X_L; 10 nM A11	1:3:4	94.69 ± 7.52	2.83 ± 0.22	5
α7 + NACHO + Bcl-X_L; 100 nM A11	1:3:4	37.13 ± 1.47	0.92 ± 0.04	5
α7 + NACHO + Bcl-X_L; 1 µM A11	1:3:4	35.91 ± 1.88	0.93 ± 0.05	5

Mean fluorescence intensity values were calculated from individually transfected wells, with plates being imaged on the OperaPhenix or Operetta high-content imaging systems. Data are grouped by experiment, meaning the same transfection conditions may have yielded different experimental fluorescence values based on image acquisition settings and other variables.

Supplementary References

1. Sattler M, et al. Structure of Bcl-xL-Bak peptide complex: recognition between regulators of apoptosis. Science 275, 983-986 (1997).
2. Lee EF, et al. Crystal structure of ABT-737 complexed with Bcl-xL: implications for selectivity of antagonists of the Bcl-2 family. Cell Death Differ 14, 1711-1713 (2007).
3. Tao ZF, et al. Discovery of a Potent and Selective BCL-XL Inhibitor with in Vivo Activity. ACS Med Chem Lett 5, 1088-1093 (2014).
4. Kotschy A, et al. The MCL1 inhibitor S63845 is tolerable and effective in diverse cancer models. Nature 538, 477-482 (2016).
5. Unwin N. Refined structure of the nicotinic acetylcholine receptor at 4A resolution. J Mol Biol 346, 967-989 (2005).
