# Supplementary material for: α7 nicotinic acetylcholine receptor upregulation by anti-apoptotic Bcl-2 proteins
Source: Nat Commun. 2019 Jun 21;10:2746. doi: 10.1038/s41467-019-10723-x (PMC6588605; doi:10.1038/s41467-019-10723-x)
Supplement: Supplementary file 3 — Reporting Summary [file 41467_2019_10723_MOESM3_ESM.pdf]

nature research

Corresponding author(s): David S. Bredt

Last updated by author(s): May 22, 2019

# Reporting Summary

Nature Research wishes to improve the reproducibility of the work that we publish. This form provides structure for consistency and transparency in reporting. For further information on Nature Research policies, see Authors & Referees and the Editorial Policy Checklist .

## Statistics

For all statistical analyses, confirm that the following items are present in the figure legend, table legend, main text, or Methods section.

| n/a | Confirmed | |
|---|---|---|
| ☐ | ☒ | The exact sample size (*n*) for each experimental group/condition, given as a discrete number and unit of measurement |
| ☐ | ☒ | A statement on whether measurements were taken from distinct samples or whether the same sample was measured repeatedly |
| ☐ | ☒ | The statistical test(s) used AND whether they are one- or two-sided<br>*Only common tests should be described solely by name; describe more complex techniques in the Methods section.* |
| ☒ | ☐ | A description of all covariates tested |
| ☐ | ☒ | A description of any assumptions or corrections, such as tests of normality and adjustment for multiple comparisons |
| ☐ | ☒ | A full description of the statistical parameters including central tendency (e.g. means) or other basic estimates (e.g. regression coefficient) AND variation (e.g. standard deviation) or associated estimates of uncertainty (e.g. confidence intervals) |
| ☐ | ☒ | For null hypothesis testing, the test statistic (e.g. *F*, *t*, *r*) with confidence intervals, effect sizes, degrees of freedom and *P* value noted<br>*Give P values as exact values whenever suitable.* |
| ☒ | ☐ | For Bayesian analysis, information on the choice of priors and Markov chain Monte Carlo settings |
| ☒ | ☐ | For hierarchical and complex designs, identification of the appropriate level for tests and full reporting of outcomes |
| ☒ | ☐ | Estimates of effect sizes (e.g. Cohen's *d*, Pearson's *r*), indicating how they were calculated |

*Our web collection on statistics for biologists contains articles on many of the points above.*

## Software and code

Policy information about availability of computer code

| | |
|---|---|
| Data collection | Electrophysiology: pClamp 10 (Molecular Devices)<br>Immunocytochemistry: Harmony 4.8 (Perkin Elmer)<br>FLIPR calcium flux: Screenworks 4.0 (Molecular Devices)<br>Epibatidine binding: TopCount NXT (Perkin Elmer) |
| Data analysis | Electrophysiology: pClamp 10 (Molecular Devices)<br>Immunocytochemistry: Columbus 2.7 (Perkin Elmer)<br>FLIPR calcium flux: Screenworks 4.0 (Molecular Devices) |

For manuscripts utilizing custom algorithms or software that are central to the research but not yet described in published literature, software must be made available to editors/reviewers. We strongly encourage code deposition in a community repository (e.g. GitHub). See the Nature Research guidelines for submitting code & software for further information.

## Data

Policy information about availability of data

All manuscripts must include a data availability statement. This statement should provide the following information, where applicable:

- Accession codes, unique identifiers, or web links for publicly available datasets
- A list of figures that have associated raw data
- A description of any restrictions on data availability

The data that support the findings of this study are available from the authors upon request. Please see author contributions for specific data sets.

# Field-specific reporting

Please select the one below that is the best fit for your research. If you are not sure, read the appropriate sections before making your selection.

[x] Life sciences    [ ] Behavioural & social sciences    [ ] Ecological, evolutionary & environmental sciences

For a reference copy of the document with all sections, see nature.com/documents/nr-reporting-summary-flat.pdf

# Life sciences study design

All studies must disclose on these points even when the disclosure is negative.

| | |
|---|---|
| Sample size | Sample sizes were chosen based on the variability of data expected to be generated by a particular experimental technique. |
| Data exclusions | Of the experiments from which our datasets were generated, no data were excluded from analysis. |
| Replication | All findings reported in the manuscript were replicated in multiple experiments, occurring at different passage numbers for cultured cells or from different tissue samples from primary neurons. |
| Randomization | In our study, samples (cells) were allocated into different experimental groups based on the cDNA plasmids they were transfected with or the chemicals they were incubated with. |
| Blinding | Investigators were not blinded to group allocation, as results were primarily quantified using automated high-content imaging systems that minimize bias in the outcome. |

# Reporting for specific materials, systems and methods

We require information from authors about some types of materials, experimental systems and methods used in many studies. Here, indicate whether each material, system or method listed is relevant to your study. If you are not sure if a list item applies to your research, read the appropriate section before selecting a response.

## Materials & experimental systems

| n/a | Involved in the study | |
|---|---|---|
| [ ] | [x] | Antibodies |
| [ ] | [x] | Eukaryotic cell lines |
| [x] | [ ] | Palaeontology |
| [x] | [ ] | Animals and other organisms |
| [x] | [ ] | Human research participants |
| [x] | [ ] | Clinical data |

## Methods

| n/a | Involved in the study | |
|---|---|---|
| [x] | [ ] | ChIP-seq |
| [x] | [ ] | Flow cytometry |
| [x] | [ ] | MRI-based neuroimaging |

## Antibodies

**Antibodies used**

Bcl-2 (Cell Signaling Technology, host species rabbit, clone D17C4, Cat. #3498)
Bcl-XL (Cell Signaling Technology, host species rabbit, clone 54H6, Cat. #2764)
Bcl-W (Cell Signaling Technology, host species rabbit, clone 31H4, Cat. #2724)
Mcl-1 (Cell Signaling Technology, host species rabbit, clone D2W9E, Cat. #94296)
HA, DyLight 650-conjugated (Invitrogen, host species mouse, clone 2-2.2.14, Cat. #26183-D650)
HA, DyLight 488-conjugated (Invitrogen, host species mouse, clone 2-2.2.14, Cat. #26183-D488)
GluA1, N-terminal (Millipore Sigma, host species mouse, clone RH95, Cat. # MAB2263)
Anti-Rabbit IgG (H+L), DyLight 650-conjugated (Invitrogen, host species goat, polyclonal, Cat. #84546)
Anti-Mouse IgG (H+L), AlexaFluor 555-conjugated (Invitrogen, host species goat, polyclonal, Cat. # A-21422)

**Validation**

Primary antibodies were validated by detection of immunofluorescence labeling from HEK293T cells transfected with cDNA encoding the target protein or epitope, whereas no such labeling was observed from untransfected cells.

## Eukaryotic cell lines

Policy information about cell lines

**Cell line source(s)**

HEK293T cells were obtained from ATCC (catalogue number CRL-11268).

| | |
|---|---|
| Authentication | Cells were authenticated based on observation of their morphology. |
| Mycoplasma contamination | Testing for mycoplasma contamination was not performed during the course of our study. |
| Commonly misidentified lines (See ICLAC register) | *Name any commonly misidentified cell lines used in the study and provide a rationale for their use.* |
