# Supplementary material for: α7 nicotinic acetylcholine receptor upregulation by anti-apoptotic Bcl-2 proteins
Source: Nat Commun. 2019 Jun 21;10:2746. doi: 10.1038/s41467-019-10723-x (PMC6588605; doi:10.1038/s41467-019-10723-x)
Supplement: Supplementary file 4 — Description of Additional Supplementary Files [file 41467_2019_10723_MOESM4_ESM.pdf]

**Description of Additional Supplementary Files**

File Name: Supplementary Data 1

Description: List of oligonucleotide primers used for site-directed mutagenesis in this study. The corresponding cDNA target is indicated. The primer name indicates whether each primer was forward (Fwd) or reverse (Rev) in its direction of DNA replication.
